# Importance of shoulder girdle and finger flexor muscle endurance in advanced male climbers

**DOI:** 10.3389/fspor.2024.1410636

**Published:** 2024-06-28

**Authors:** Paweł Draga, Robert Rokowski, Alexander Sutor, Dominik Pandurevic, Michail Lubomirov Michailov

**Affiliations:** ^1^ Institute of Measurement and Sensor Technology, UMIT-Private University for Health Sciences, Medical Informatics and Technology GmbH, Hall in Tirol, Austria; ^2^ Department of Tourism and Leisure, Section of Mountaineering and Qualified Tourism, University of Physical Education, Krakow, Poland; ^3^ Department Theory and Methodology of Sports Training, National Sports Academy “Vassil Levski” Sofia, Sofia, Bulgaria

**Keywords:** sport climbing, muscles endurance, specific fitness tests, absolute and relative endurance, Edlinger test

## Abstract

**Aim:**

This study aimed to: (a) assess the relationships between climbing performance and finger and shoulder girdle muscle endurance; and (b) provide evidence on the validity of the specialized exercise tests used for the purpose.

**Materials and methods:**

28 male sport climbers (climbing ability 23 ± 2.43 IRCRA scale) performed four tests muscle failure, including two-finger hang tests (using 2.5 and 4 cm holds) and two variants of pull-up exercises (classical pull-ups and a combination of dynamic and isometric actions – the so-called Edlinger). Climbing performance and test results were subjected to correlation, taxonomic and regression analysis.

**Results:**

The correlations between the results from all tests and climbing performance were notably strong (r between 0.54 and 0.61) and statistically significant (p<0.05). The taxonomic analysis indicated that the two variants of each test type reflect two different latent variables 2.5 cm and 4 cm finger hang durations were highly correlated (r=0.76,p<0.01). A similar correlation was found between the results from the pull-up tests (r=0.72,p<0.01). Thus, the finger hang and pull-up test results were determined to a high extent (43% and 49%, respectively) by factors that cannot be assessed when only one test of each type is used. The regression model of the two-finger tests allowed individual endurance profiles to be assessed.

**Conclusions:**

The muscular endurance of the elbow flexors and shoulder girdle muscles predicts climbing performance within the specific sport level studied to a comparable degree as finger flexor endurance.The use of two variants of a test intended to assess one physical ability provided important details on a climber’s fitness.

## 1 Introduction

Sport climbing is a discipline that requires both endurance and strength, combined with complex biomechanics. During climbing, climbers overcome their body weight. In overhanging terrain, the body weight is distributed predominantly to the upper limb supports (1). The finger flexor muscles are responsible for holding the upper limb supports while acting isometrically. Considerable efforts are also made by the elbow flexor and shoulder girdle muscles. They largely contribute through both dynamic and isometric actions for maintaining the so-called lock-off positions and for moving the center of mass forward on the route or closer to the wall. As they are relatively small, the finger flexors followed by the shoulder girdle muscles exert a higher percentage of their maximal voluntary contraction and experience deeper fatigue compared to other muscle groups (2, 3). Furthermore, the main load characteristics during climbing are the high-intensity intermittent isometric efforts with an unfavorable ratio of finger flexor muscle contraction and relaxation phases: 6.3:1.5 s in modern lead climbing competitions (4) and 7.9:0.6 s in bouldering competitions (5). This restricts the local blood flow and energy and oxygen supply. Thus, finger maximal strength and the ability to maintain high force for longer, as well as local forearm muscle aerobic and anaerobic capacity are factors of major importance in climbing. This was proven by many researchers who used maximal strength and continuous or intermittent endurance tests (6–10). Although fewer, studies on shoulder girdle strength and endurance succeeded in demonstrating that more advanced climbers have greater shoulder girdle strength, power, and endurance than less skilled climbers or non-climbers (11–17).

The load during climbing places high demands not only on finger and shoulder girdle muscle strength and endurance but also on other physical characteristics. Success in climbing depends on sport-specific hip mobility (18), and body composition elements such as low body mass (19) 66.1±6.40 kg, reduced body fat (20) 10.5±5.08 %, and average height(21) 175.6±11.0 cm.

To optimally direct training, climbers and coaches should be aware of the relative importance of finger and shoulder strength and endurance as key performance factors. Rokowski et al. (22) calculated correlation coefficients between various physical variables and climbing performance. The highest correlation was observed for maximal finger strength relative to body mass (r=0.71), followed by finger endurance (r=0.68). The upper limb index [body height/arm length] also showed a significant correlation (r=0.66). In this studies, it was noted that less important factors included maximum finger strength and the maximum number of pull-ups on the bar.

The same author (23) used a climbing–specific test known as ”Edlinger's alphabet” performed on a bar and including a combination of dynamic and isometric upper limb actions, which again correlated with climbing level (*r* = 0.51). Grant (14) showed that elite climbers performed significantly better than recreational climbers and non-climbers when compared by the results from a finger strength test and the number of pull-ups and bent arm hang durations. Unfortunately, the effect sizes for estimating the relative importance of the qualities assessed through these later tests were not provided. Michailov and Baláš (24) found that finger strength correlated stronger with climbing performance than shoulder girdle strength (r=0.81 vs. r=0.65). Baláš et al. (11) showed that the finger hang determines to a greater extent climbing performance than bent-arm hang only in men ($r^{2}=0.76$ vs. $r^{2}=0.49$ in men and $r^{2}=0.66$ vs. $r^{2}=0.64$ in women).

The tests used by the researchers in climbing can be generally described as simple and dynamometric. Simple tests are for example finger hangs, bent-arm hangs, pull-ups, combinations between bent-arm hangs and pull-ups (e.g., Edlinger test), and others (11, 14, 23, 25). The simple test scores performed until muscle failure can be considered absolute endurance measures. They depend not only on finger endurance but also on strength and body mass. The combined effects of these three factors lead to strong correlations between hanging time and climbing performance (11, 26). To be able to better optimize and individualize training based on testing, relative endurance measures should be used. Such measures are the results of dynamometric tests performed at the same relative intensity assigned as a percentage of the climber’s maximal voluntary contraction. A common and established way of performing such tests involves one-arm finger hangs with feet on the ground, using the body weight to load the hold and real-time feedback to be able to apply and maintain the target force (16, 26). Nevertheless, simple tests can easily be reproduced by many climbers and also allow modeling conditions. For example, Rokowski and Staszkiewicz (23) used 2.5 cm and 4 cm edges during finger hang testing.

It should be noted that the criterion validity (in terms of specificity and correlations with climbing performance) of the above-mentioned tests was determined. However, construct validity evidence (the extent to which a test assesses muscle strength or endurance) was not provided for most of these tests. Many of these studies have been conducted using different tests performed by groups with different levels of climbing performance. This has led to inconsistent and incomparable results reported by different researchers. Furthermore, existing studies on shoulder girdle endurance in sport climbing are limited in scope. The majority of researchers have focused on the assessment of strength and endurance of the finger flexors and it is still not clear whether shoulder girdle and elbow flexor endurance are equally important as that of the finger flexors.

There is a need to develop the most useful and simplest tests possible for a wide range of climbing practitioners and theorists. Using simple tests with different maximum durations can allow for the assessment of relative endurance without the need for expensive equipment. The introduction of the Edlinger test into research is justified due to its variety of contractions, including concentric, eccentric, and 7-s isometric contractions. The distinguishing factor of this test is its combination of different types of contractions, making it interesting compared to bent-arm hang. Additionally, it is important to compare this test with the classic pull-up. Therefore, the aim of this study: (a) to assess the relationship between climbing performance and the endurance of finger and shoulder girdle muscles; and (b) to provide evidence of the validity of two finger endurance tests and two shoulder girdle endurance tests.

## 2 Materials and methods

Twenty-eight male climbers participated in the study, all at the actual sport level corresponding to the IRCRA 19 – 27 Red-point scale (27). The study group exhibited the following characteristics: an average age of 28.4±6.08 years, an average body weight of 70.13±5.31 kg, an average body height of 178.14±5.80 cm, and a minimum duration of sports practice of 3 years.

Ethical considerations were strictly followed, and the study adhered to the recommendations of the local Research Ethics Committee in line with the principles outlined in the Declaration of Helsinki (28). All participants were provided with comprehensive information about the potential risks associated with the experiments and gave their informed consent before data collection. The effect of hold size is an important determinant of effort duration and can involve different metabolic processes in the muscles of climbers at different levels of advancement (23). The relationship of muscular endurance tested with different hold sizes should be verified; for this reason, holds of 2.5 and 4 cm were used in this study.

**Figure 1:**
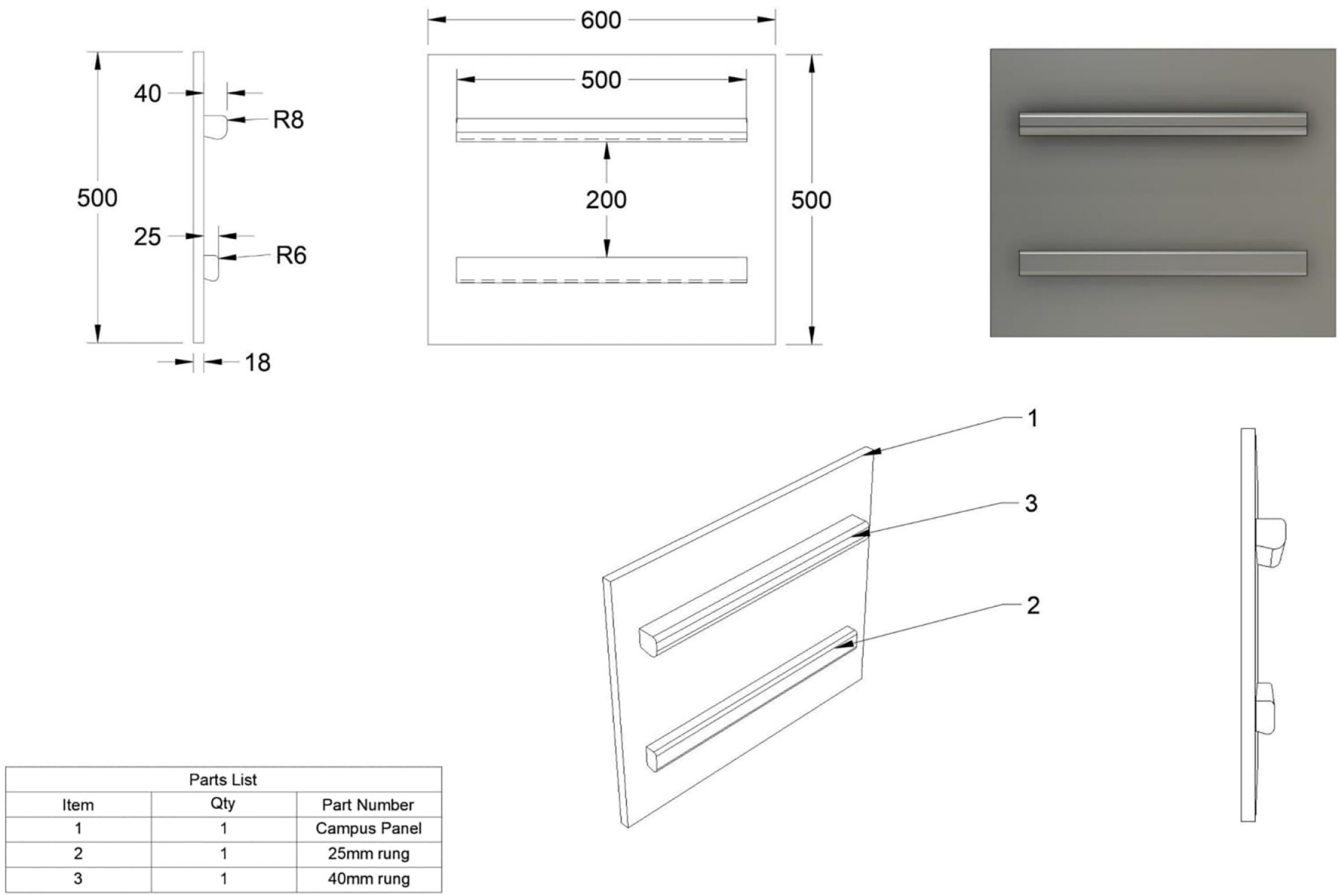
Campus board.

**Figure 2:**
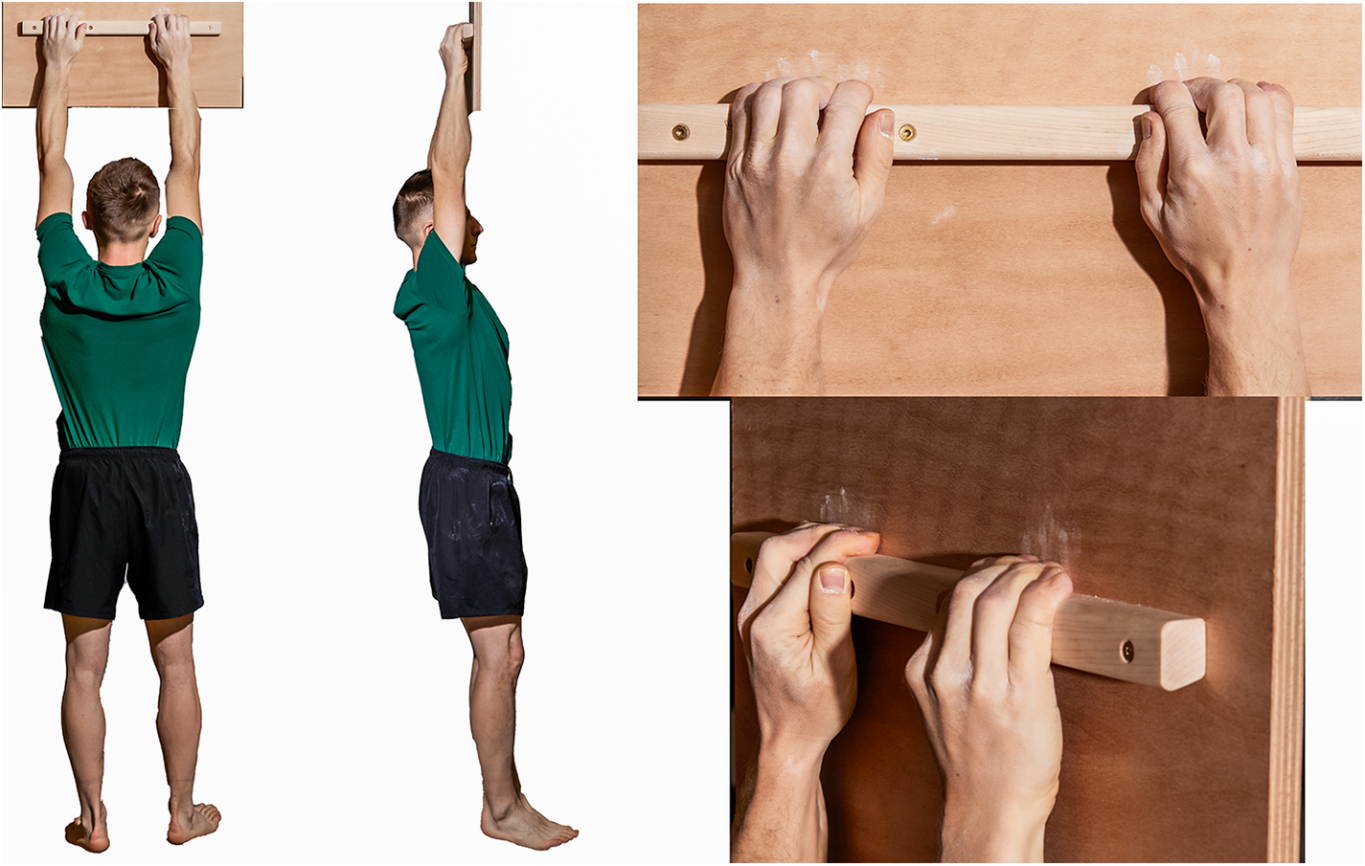
Hang 2.5 - hang with both hands on a hold 2.5 cm deep until failure.

**Figure 3:**
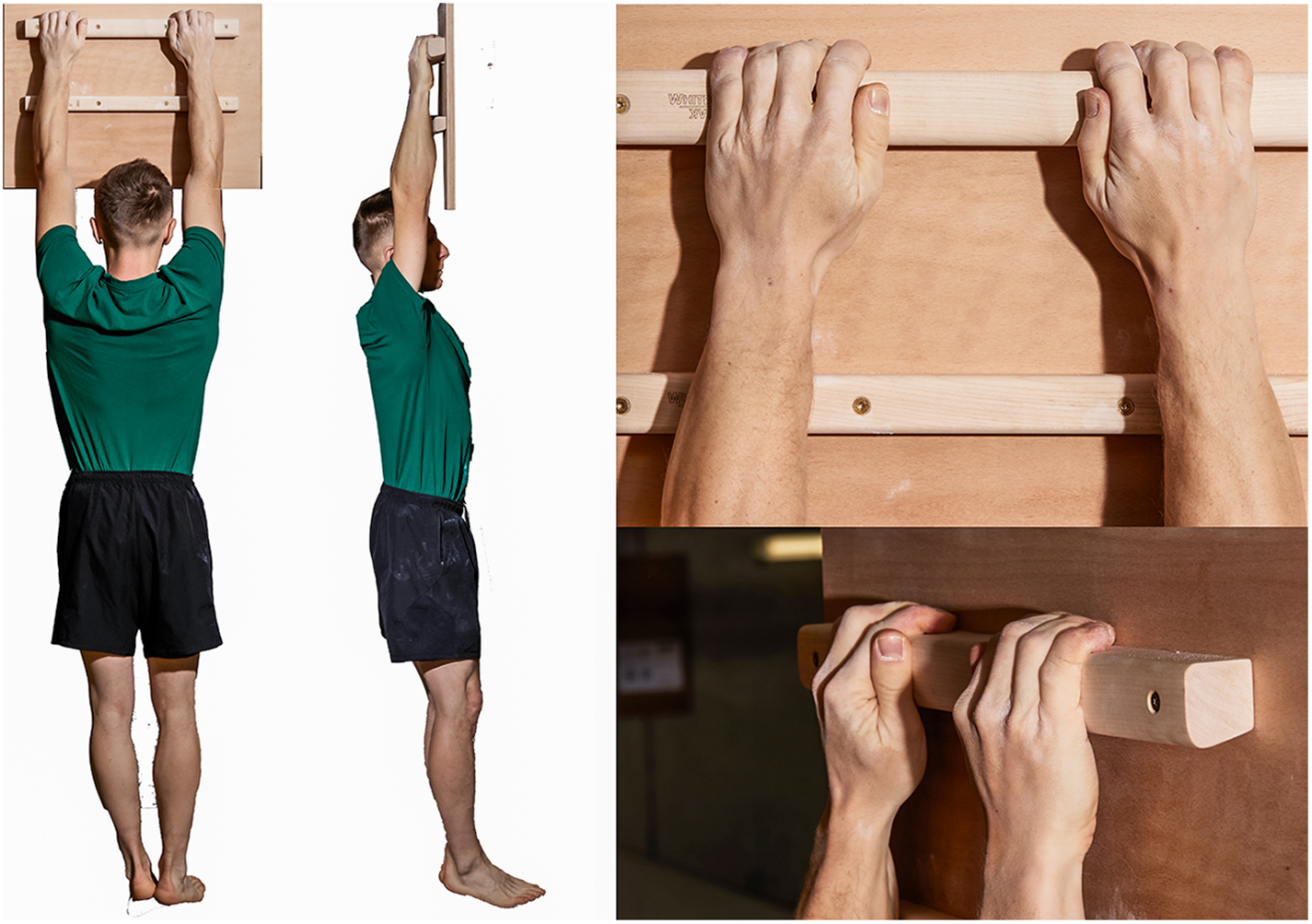
Hang 4 - hang with both hands on a grip 4 cm deep until failure.

**Figure 4:**
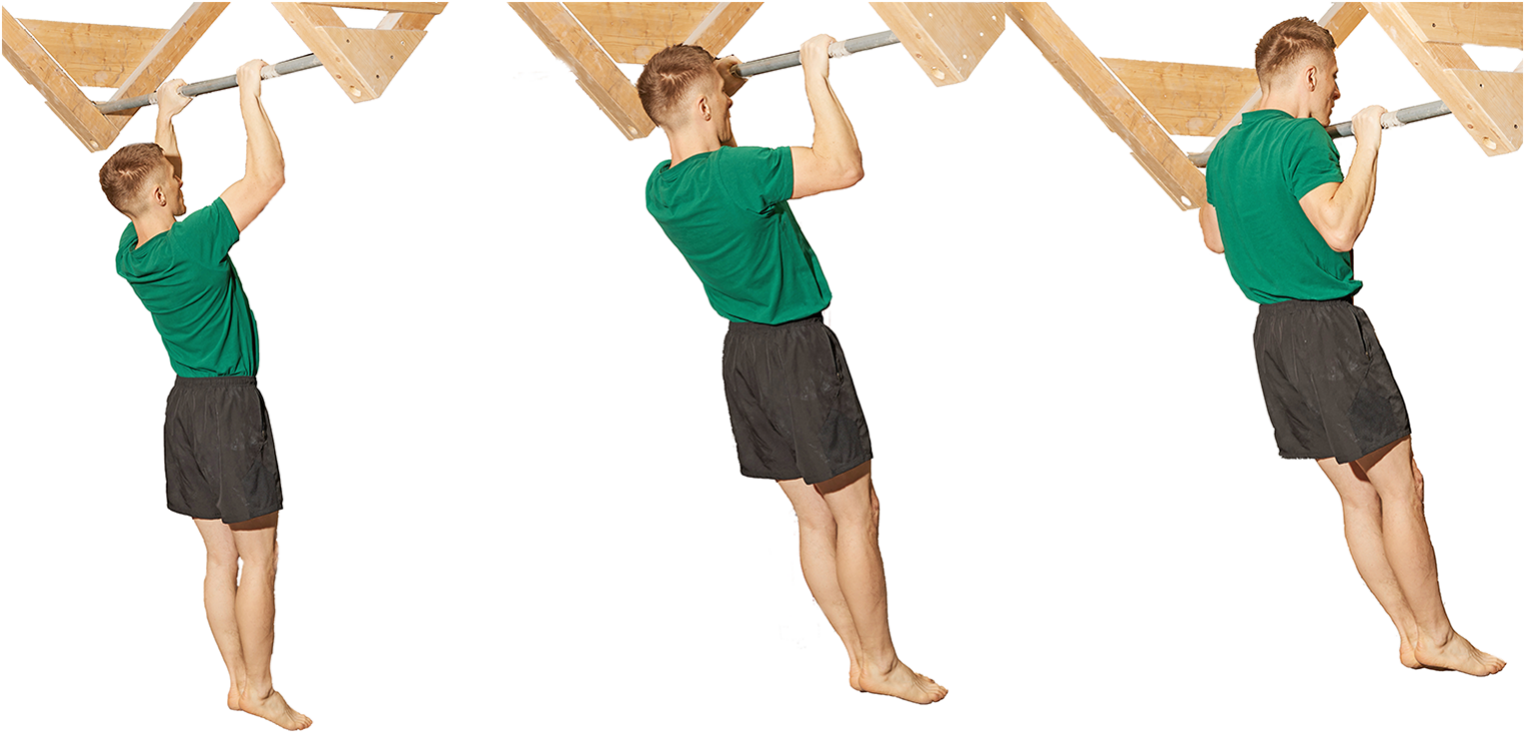
The Edlinger test (29).

Specific physical fitness tests were conducted using a measuring device called a campus board, which was mounted at a $90^{\circ }$ angle to the ground and included two test holds with depths of 2.5 cm and 4 cm, and a width of 50 cm (Figure 1). For clarity and simplicity, these tests were given working names. Muscle endurance was assessed using the following tests:

1. Finger hang 2.5 (Figure 2): In this trial, the subjects were required to hang with both hands on a hold that was 2.5 cm deep. The subjects gripped the ledge with all four fingers of each hand (excluding the thumb, and open grip), maintaining their hands at shoulder width, with their upper extremities fully extended, and their bodies hanging vertically. The test measured the duration for which the subjects could sustain this position, with a precision of 1 s.
2. Finger hang 4 (Figure 3): Similar to the hang 2.5 test, this trial involved hanging with both hands on a hold, but the hold in this case was 4 cm deep. The same parameters for grip and body position were maintained, and the test measured the duration of this hang with a 1-s precision.
3. Edlinger test (29) (Figure 4): This test involved a series of cycles in which the subject performed two pull-ups on a bar. After the second pull-up, they held their chin over the bar for 7 s (cycle I). Subsequently, the subject repeated this process with two more pull-ups and held their elbows at a $90^{\circ }$ angle (cycle II). This cycle was repeated, increasing the angle of elbow flexion in each subsequent cycle (III, IV, V, and so forth).
4. Pull-up test: this test measured the maximum number of pull-ups a given climber could perform on the bar. The test followed the following rules: Participants performed pull-ups on a standard horizontal bar. They had to lift their bodies from a position of full shoulder extension, hanging with a pronated grip at shoulder width, until their chins touched the bar. The rhythm of the pull-up was not regulated. During the test, it was forbidden to take the hands off the bar and use leg movements to support the pull-up by swinging.

Statistical calculations were carried out on the measured data, resulting in the determination of arithmetic means (x), standard deviations (SD), minimum (min), and maximum (max) values, Pearson’s linear correlation coefficients, and a regression model. The French difficulty scale, an ordinal scale with no SI units, was used to assess climbing levels. To enable statistical analysis, a conversion was made from the French scale to the IRCRA point scale, according to Draper (27). The correlation coefficients were calculated to determine the relationships between the tests as well as the influence of the measured abilities on climbing performance. Additionally, taxonomic analysis was used to provide construct validity evidence and assess the significance of climbers’ endurance tests. The estimated regression model was used to further analyze the relationship between finger hang test scores and to propose an approach for the assessment of climbers’ endurance profiles based on Z-scores. Z-scores were computed using the model’s equation, climber’s actual finger hang 2.5 duration and a formula including the actual (y) and predicted (Y) finger hang 4 durations and the standard error of the estimate $S_{YX}$:

$$Z=\frac{y-Y}{S_{YX}}$$

All calculations were performed using StatSoft Statistica 13.3 software.

## 3 Results

Table 1 presents the numerical characteristics of the endurance tests conducted on elbow flexor and shoulder girdle muscles. The collected data reveal considerable variability in the parameters measured. Notably, the results from the hang tests on the 2.5 cm and 4 cm hold exhibited a substantial difference of nearly 300% in the duration of effort between the highest (27) and lowest (19) ranked athletes (13), considering the Red-point style climbing level. Additionally, the Edlinger test, which incorporates concentric contractions, eccentric contractions, and 7-s static phases at specific angles, underscored a noticeable disparity in the number of cycles completed, ranging from 12 cycles for the highest-ranked climber to 3 cycles for the lowest-ranked climber.

**Table 1:** Basic descriptive statistics of the variable endurance parameters.

Variable	N	Mean	SD	Min–Max
IRCRA	28	23.00	2.43	19–27
Hang 2.5 (s)	28	61.70	17.29	33–103
Hang 4 (s)	28	90.13	23.21	45–133
Edlinger (rep)	28	6.90	1.97	3–12
Pull-ups (rep)	28	25.40	7.83	11–40

The study found significant correlations between assessment for all tests and sports performance. Pearson correlation coefficients, presented in Table 2, showed the strongest correlations for variables related to elbow flexor muscle strength, in particular pull-ups (r=0.60) and the Edlinger test (r=0.60). Tests involving the endurance of the finger flexor muscles also showed a correlation with the climbers’ sports performance (hang 2.5 r=0.54 and hang 4 r=0.57). The study demonstrated significant intercorrelations between tests assessing shoulder girdle strength (r=0.72) and tests assessing finger flexor strength (r=0.76). There were also intercorrelations between shoulder girdle tests and finger flexor tests but with significantly lower values (Table 2).

**Table 2:** Pearson’s linear correlations (r) of motor variables with the performance level of climbers.

Variable	Climbing level	
	r	p-value
Hang 2.5	0.54∗	0.003
Hang 4	0.57∗	≪ 0.001
Edlinger	0.60∗	≪ 0.001
Pull-ups	0.61∗	≪ 0.001

Correlations significant at p<0.05 are highlighted with ∗, n=28.

**Figure 5:**
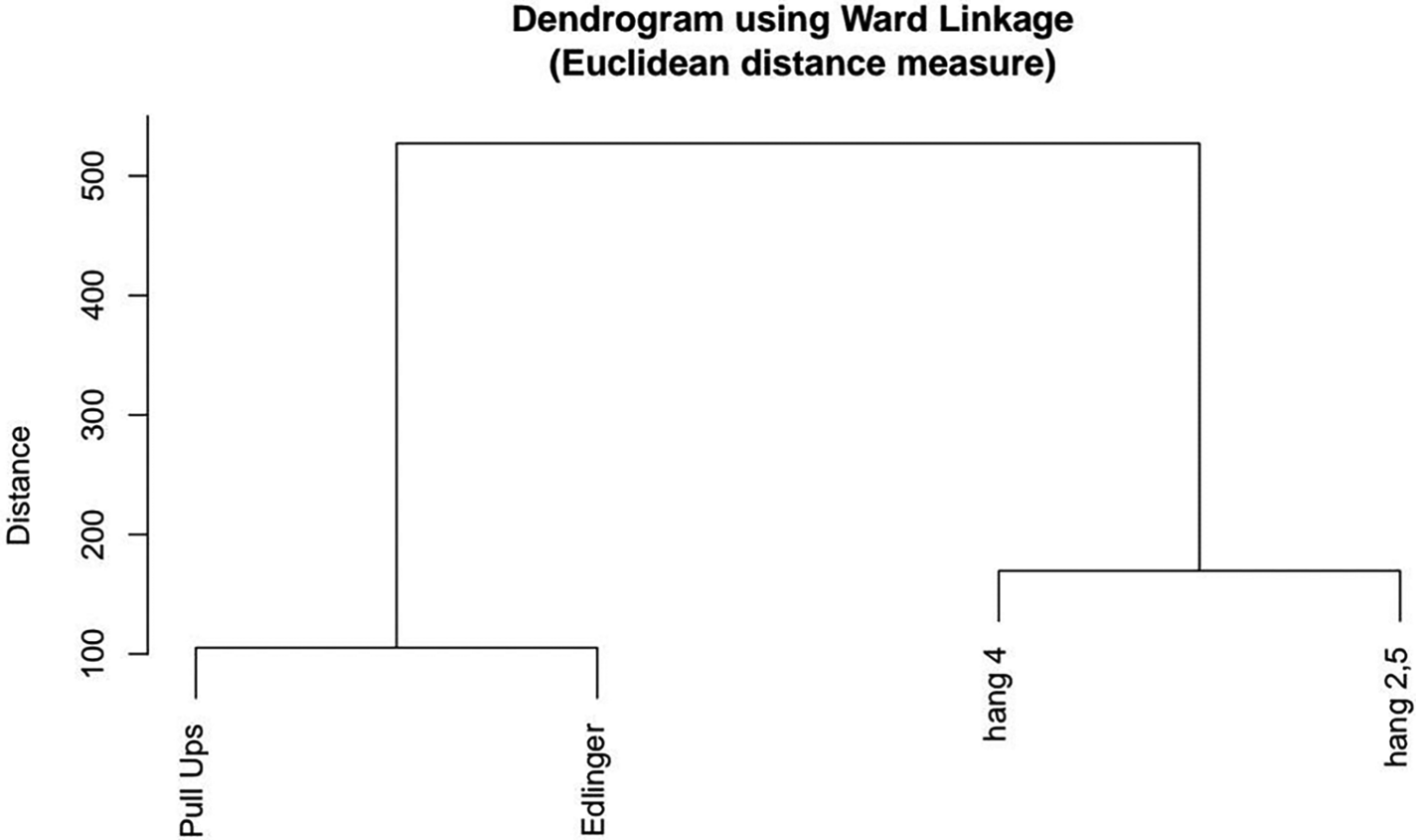
Ward’s tree diagram of climbers’ motor abilities: (1) Pull Ups-endurance of the arm muscles, (2) Edlinger - endurance of the arm muscles (3) Hang 4 - endurance of the finger muscles of a 4 cm hold, (4) Hang 2.5 - endurance of the finger muscles of a 2.5 cm hold.

The taxonomic analysis, depicted in the Ward tree diagram (Figure 5), revealed the interconnections of the hang tests at different depths of holds, specifically hang 2.5 and hang 4, placing them within the same factor group. Following the hypothesis, the maximum number of pull-up tests was also positioned in the tree diagram in conjunction with the Edlinger test. The findings indicate that the hang tests are indicative of finger muscle endurance, while the other tests are indicative of arm endurance. Moreover, the dendrogram showed minimal Euclidean distances between the tests, confirming significant correlations between the tests performed (Table 3).

**Table 3:** Intercorrelations (r) of motor variables.

Variable	Hang 2.5		Hang 4		Edlinger		Pull-ups	
	r	p-value	r	p-value	r	p-value	r	p-value
Hang 2.5	–	–	0.76∗	≪ 0.001	0.46∗	0.013	0.32	0.097
Hang 4	0.76∗	≪ 0.001	–	–	0.52∗	0.0045	0.40∗	0.035
Edlinger	0.46∗	0.013	0.52∗	0.0045	–	–	0.72∗	≪ 0.001
Pull-ups	0.32	0.097	0.40∗	0.035	0.72∗	≪ 0.001	–	–

Correlations significant at p<0.05 are highlighted with ∗, n=28.

**Figure 6:**
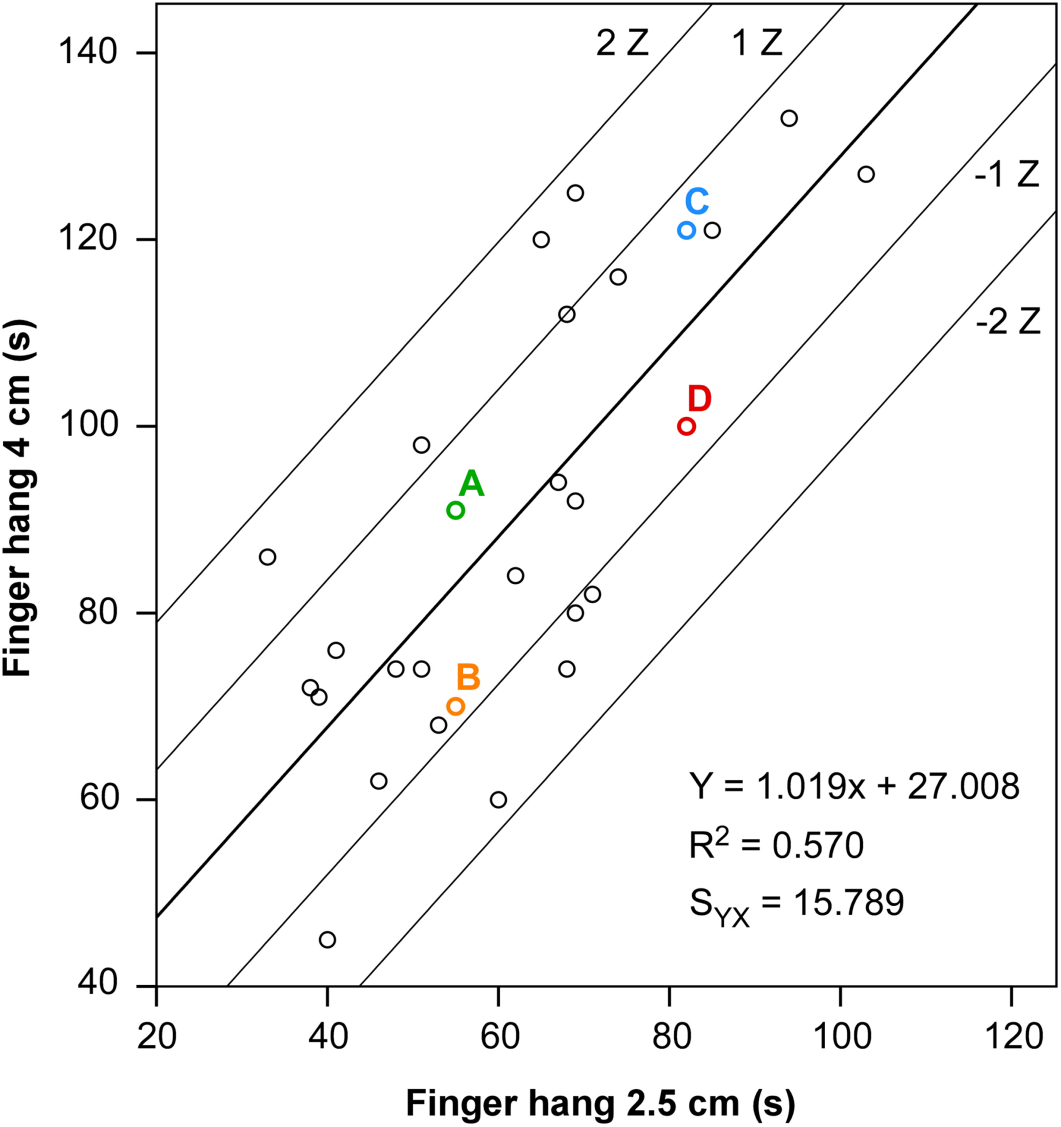
Regression model of the relationship between test durations from the finger hang 4 cm and finger hang 2.5 cm. The tick line represents the regression model’s line. The thin lines are built based on the standard error of estimate and represent Z-score limits. The colored data points are examples of climbers with different relative endurance levels. **Examples:***Climber A and climber B possess lower absolute endurance levels than climber C and climber D. Moreover, Climber A and climber B as well as climber C and climber D achieved the same finger hang 2.5 test results (55 s and 82 s, respectively). However, climber A* (Z=0.502) *has higher relative endurance than climber B* (Z=−0.828) *and climber D* (Z=−0.671). *Climber A and Climber C* (Z=0.659) *have close relative endurance levels.*

The linear regression model of the relationship between finger hang test durations is presented in Figure 6. The finger hang 4 duration as a dependent variable was 57% determined by the finger hang 2.5 duration and 43% by other factors. The standard error of the estimate was considerable (16 s). However, the model was adequate (F=34.434, p<0.001) and the parameters of the equation were significant (slope = 27.008, p=0.023; intercept = 1.019, p<0.001). The significance of the model enabled the calculation of Z-scores reflecting the deviation of the actual from the predicted finger hang 4 results. Thus, examples of climbers with different relative endurance were given.

## 4 Discussion

The present study found that both finger flexor and shoulder girdle muscular endurance were significant factors in advanced climbing, as shown by the correlation analysis. This reinforces findings in a limited number of previous studies that collected data on the endurance of both muscle groups in climbers (11, 25). To the best of our knowledge, this is the first study that uses more than one finger flexor and shoulder girdle test to provide construct validity evidence. The taxonomic analysis clustered the tests into two types and showed that none of the tests is more suitable (compared to the other test from its type) for application in climbing. The present study proposes a new simple approach for the assessment of climbers’ relative endurance based on two easy-to-organize finger hang tests with different hold sizes and durations.

### 4.1 Elbow flexor and shoulder girdle muscle endurance

The maximum number of pull-ups and the Edlinger test results significantly correlated with the climbing performance. The climber with the highest climbing ability (27 IRCRA) showed a significant advantage over the lowest-ranked climber (19 IRCRA) (29 vs. 11 pull-ups). Nevertheless, the correlation cannot be characterized as very strong (r between 0.54 and 0.61). A study by Ozimek et al. (25) confirms that the endurance of the elbow flexor and the shoulder girdle muscles influences climbing performance to a similar degree as the endurance of the finger flexor muscles. Devise et al. (30) also showed a significant correlation between the number of pull-ups and climbing levels (r>0.39). The researchers investigated athletes with similar sports achievements as the participants in the present study and obtained similar minimum, maximum, and mean values (11, 40, and 23 pull-ups, respectively). Thus, the pull-up test on a bar appears to be a useful diagnostic tool to assess muscular endurance in sport climbing.

It should be noted that the performance of the pull-up test has a standardization issue. In multiple pull-ups with the arm in a pronated position, the elbow and shoulder joints flexed in a range of 96.8±17.2 and 84.0±17.8 degrees, respectively (31). In a single pull-up, these angles were 110.1±10.9 and 100.3±13.2 degrees, respectively. Devise et al. (30) found that the control of movement speed, transition from eccentric to concentric stretch-short cycle (SSC) phase, grip width, hand position, and lower limb work played an important role in the performance of the pull-up test. In addition, LaChance and Hortobagyi (32) and Vigouroux et al. (31) found that the use of rhythm and SSC can influence test results by up to +20% per number of reps.LaChance and Hortobagyi (32) found that the number of pull-up repetitions is significantly affected by cadence because it interacts with intramuscular occlusion.

In their study, Winkler et al. (4) measured the average reach phase and contact times of the hand in the lead events for athletes in the qualification and semi-final rounds of international competition. During the qualifying phase, the contact time was 8.01 s for women and 6.61 s for men, and the reach-to-hold time was 2.17 s (women) and 2.07 s (men). In the final round, both contact and reach-to-hold times decreased significantly for both sexes. Therefore, muscle time under tension (TUT) is significant at different arm angles, indicating the need to include this factor in testing procedures. The Edlinger test meets these requirements. Correlations were noted between the pull-up and the Edlinger test (r=0.72). This means that the outcome of one test depends 51% on the measures included in the other test. Other unaccounted factors, which have a value of 49%, should include the speed and time of the test, as well as the effect of the isometric phase used in the Edlinger test.

The similarity of the two shoulder girdle tests is also reflected by the use of the Ward tree diagram method. It indicates that both tests belong to a group that measures the same latent variable and neither of the tests is to be preferred. Nevertheless, the Edlinger test is more specific to the requirements of the discipline because of the 7-s isometric phases within the three stages of the pull-up, at angles of 120, 90, and 30 degrees elbow flexion. Interestingly, Rokowski and Tokarz (33) found that Edlinger test results correlate with climbing performance in elite (r=0.51) but not beginner climbers. The mean values for the elite and beginning groups were 6.8±1.5. and 3.5±1.5. Cycles, respectively. In addition, Michailov et al. (10) found that advanced climbers sustained longer than non-climbing controls during isolated continuous elbow flexor contractions at intensities of 70% and 50% maximum voluntary contraction (MVC). However, unlike elbow flexor strength (relative to body mass), the results from these endurance tests did not correlate significantly with climbing performance. The above-mentioned suggests that the Edlinger test should be verified as a diagnostic tool with climbers of different climbing ability. The effect of arm strength on the results of this test is also unknown and should be verified by future studies.

### 4.2 Finger flexor muscle endurance

There were minor differences between the strength of the relationships between climbing performance and finger and shoulder girdle test results. It appears that finger and shoulder girdle strength are equally important in advanced climbing. There were also minor differences between the correlation coefficients representing the relationships between climbing performance and the finger hangs on 2.5 cm and 4 cm hold (r=0.57 and r=0.54, respectively). Therefore, all of the four tests can be considered useful in climbing. The two finger hangs were strongly correlated (r=0.76), meaning that one of the tests determines by 57% the result obtained in the other test. The remaining 43% of the variance is due to factors that cannot be reflected when the tests are performed separately. These factors could be finger grip position or different energy system contributions during the two tests. Although the tests differed in hold size the open grip position was used in both tests. However, the duration of finger hang 2.5 was 62 s and the test should assess predominantly local muscle anaerobic capacity. Finger hang 4 duration was 90 s and its duration should depend on both anaerobic and aerobic energy delivery. The metabolic data of Maciejczyk et al. (9) collected during climbing-specific exercise tests support this statement. The relative aerobic energy contributions during a 30 s all-out, 60 s continuous and 234 s intermittent maximal efforts were 19%, 28%, and 60%, respectively. Both fingers hang 2.5 and finger hang 4 primarily assess finger flexor endurance, with only limited assessment of the shoulder girdle muscles (34). This is evidenced by the Ward tree analysis, which separated these tests from the pull-ups and the Edlinger test. In addition, the present finger hang tests were found to be affected by finger strength. Ozimek et al. (25) found that the hang performed on the smaller hold had a strong correlation with finger strength (r=0.72). This correlation was more pronounced in climbers at a higher level. However, the researchers assumed that hanging on the larger hold is more appropriate for testing climbers with different ability levels.

The results of the finger hang 2.5 and finger hang 4 tests confirm a strong relationship between finger flexor endurance and climbing performance. This conclusion has been repeatedly reported by researchers in both simple and dynamometric intermittent or continuous tests (6, 35, 36). Previously, comprehensive evaluation of climbers’ endurance was achieved by dynamometric tests assigning intensity at different % MVC. The use of two simple finger hang tests in the present study also provided details on participant’s endurance profiles (Figure 6). The external load (body weight) and type of muscle action (isometric) were the same in both tests. However, the two hold sizes induced different muscle contraction intensities. Therefore, the hanging time was shorter when using the smaller hold. The results from both tests correlated significantly. Generally, a climber with a better result in finger hang 2.5 should perform better during finger hang 4, as shown by the regression model. However, the possible deviation from the model was relatively high (16 s standard error of the estimate). The variance was explained to a considerable extent (43%) by factors that should be related to aerobic capacity and relative endurance. It was easy to identify three types of climbers’ endurance profiles: “higher relative endurance”, “lower relative endurance” and “balanced”. Climbers can be quantified based on the presented Z-score. Climbers with positive Z-scores cope better during longer efforts at a lower intensity (i.e., “higher relative endurance”). Climbers with negative Z-scores cope better during shorter efforts at a higher intensity (i.e., “lower relative endurance”). This type of evaluation clearly shows that a longer hanging time in one of the tests does not necessarily mean better relative endurance (see examples in Figure 6). Knowing the level of relative endurance can help the coach avoid making wrong conclusions about the climber’s training state. Future studies are needed to estimate the proper hold sizes for each climbing ability level and to investigate consistency between the relative endurance indicators and results from dynamometric finger endurance tests and muscle tissue oxygenation parameters.

## 5 Conclusions

Finger flexor and shoulder girdle muscle endurance are equally important key performance factors in advanced climbing. The finger hangs on 2.5 cm and 4 cm holds as well as the pull-up and Edlinger tests appeared to be valid. This was evidenced by the significant correlations between climbing performance and test results. In addition, the taxonomic analysis demonstrated that the finger hang tests should assess one latent variable (finger endurance), while the shoulder girdle tests should assess another latent variable (shoulder endurance). Thus, all tests can be considered useful for the assessment of climbers’ fitness. The combination of two finger hang tests with different maximum durations allowed the assessment of climbers’ relative endurance and most likely reflects the capacity of the different energy systems.

## 6 Practical applications

The present results demonstrate that climbers’ training should include not only exercises for developing the endurance of the forearms but also of the elbow flexors and shoulder girdle muscles. The pull-up test exhibits reliability, validity, simplicity, ease of interpretation, and safety, making it a valuable tool for assessing muscle endurance in climbers across various climbing ability levels. The Edlinger test is rarely used. However, it is climbing-specific and has the potential to diagnose mechanisms that are only partially assessed by traditional tests such as the pull-up or bent-arm hang test. The system approach related to the use of two variants of a muscle endurance test that differ in duration adds useful information about the climber’s endurance profile. This type of testing aids in the proper direction and individualization of training loads and does not require expensive equipment. The present study may prompt future research for further validating the approach for assessing relative endurance and the applicability of the Edinger test in climbers at different ability levels. During the execution of the tests, it is imperative to exercise particular caution due to the maximal nature of the exertion involved. This places significant strain on structures such as the fingers, elbows, and shoulders, which are the most frequent sites of injury in climbing (37).

## Data Availability

The original contributions presented in the study are included in the article/Supplementary Material, further inquiries can be directed to the corresponding author/s.
